# Changes in Parthenogenetic Imprinting Patterns during Reprogramming by Cell Fusion

**DOI:** 10.1371/journal.pone.0156491

**Published:** 2016-05-27

**Authors:** Hyun Sik Jang, Yean Ju Hong, Hyun Woo Choi, Hyuk Song, Sung June Byun, Sang Jun Uhm, Han Geuk Seo, Jeong Tae Do

**Affiliations:** 1 Department of Animal Biotechnology, College of Animal Bioscience and Technology, Konkuk University, Seoul, Republic of Korea; 2 Animal Biotechnology Division, National Institute of Animal Science, Rural Development Administration, Suwon, Republic of Korea; 3 Department of Animal Science and Biotechnology, Sangji Youngseo College, Wonju, Republic of Korea; Guangzhou Institute of Biomedicine and Health, CHINA

## Abstract

Differentiated somatic cells can be reprogrammed into the pluripotent state by cell-cell fusion. In the pluripotent state, reprogrammed cells may then self-renew and differentiate into all three germ layers. Fusion-induced reprogramming also epigenetically modifies the somatic cell genome through DNA demethylation, X chromosome reactivation, and histone modification. In this study, we investigated whether fusion with embryonic stem cells (ESCs) also reprograms genomic imprinting patterns in somatic cells. In particular, we examined imprinting changes in parthenogenetic neural stem cells fused with biparental ESCs, as well as in biparental neural stem cells fused with parthenogenetic ESCs. The resulting hybrid cells expressed the pluripotency markers *Oct4* and *Nanog*. In addition, methylation of several imprinted genes except *Peg3* was comparable between hybrid cells and ESCs. This finding indicates that reprogramming by cell fusion does not necessarily reverse the status of all imprinted genes to the state of pluripotent fusion partner.

## Introduction

Pluripotent stem cells can differentiate into all three germ layers *in vitro* and *in vivo*, and have unlimited capacity to self-renew [1–3]. Embryonic stem cells (ESCs), which are derived from the inner cell mass of a blastocyst, are the gold standard of pluripotency [4, 5]. Pluripotency is lost upon differentiation, but differentiated somatic cells can be reprogrammed back to the pluripotent state by nuclear transfer, cell fusion, and transduction of defined reprogramming factors. Indeed, somatic cells reprogrammed through nuclear transfer can be subsequently induced to form totipotent embryos, from which pluripotent ESCs may be derived [6]. On the other hand, ESCs, embryonic germ cells, and embryonic carcinoma cells are typically used to reprogram somatic cells by cell-cell fusion [7–9]. Finally, induced pluripotent stem cells (iPSCs) were generated from somatic cells by exogenous expression of defined transcription factors, including Oct4, Sox2, Klf4, and c-Myc [10]. These cells express pluripotency-related genes, differentiate into all three germ layers *in vitro*, generate germ-line chimeras [11], and, remarkably, confer pluripotency to somatic cells by cell-cell fusion [12].

Genomic imprinting patterns in pluripotent stem cells are distinct from those in somatic cells. Imprinted genes are expressed from a single allele according to the parent of origin, and regulate fetal and/or placental development [13–15]. Genomic imprinting is erased during migration of primordial germ cells, but reestablished during gametogenesis. In primordial germ cells, genomic imprinting and DNA methylation patterns form a gradient along the phases of migration. Notably, embryonic germ cells derived from these primordial germ cells retain the imprinting pattern present at the time the germ cells are obtained [8]. Remarkably, however, reprogramming by transduction of defined factors modifies genomic imprinting, DNA methylation, and expression of imprinted genes [16]. For instance, maternally imprinted genes, which were completely methylated in parthenogenetic somatic cells, were demethylated after reprogramming into pluripotent state [17, 18].

In this study, we investigated whether pluripotent ESCs reprogram genomic imprinting in somatic cells by fusing biparental ESCs with parthenogenetic somatic cells, and vice versa. We hypothesized that imprinting patterns of pluripotent fusion partners, ESCs or parthenogenetic ESCs (pESCs), dictate the imprinting patterns in the resulting hybrid cells.

## Materials and Methods

### Animal use ethical statement

Experiments were carried out in accordance with the approved guidelines and all experimental protocols were approved by the Institutional Animal Care and Use Committee (IACUC) of Konkuk University. All mouse strains were bred and housed at the mouse facility of the Konkuk University or were bought from Orient-Bio Inc. (Gyeonggi-do, Korea; http://www.orient.co.kr). Animal welfare was under control of local committees. Mice were housed in a temperature-controlled room with automated darkness-light cycle system, fed with a regular ad libitum feeding. Before oocyte harvesting, mice were sacrificed by carbon dioxide inhalation.

### Generation of parthenogenetic embryonic stem cells

B6D2F1 mice were induced to superovulate by serial injections of 10 IU pregnant mare serum gonadotropin and 12 IU human chorionic gonadotropin 48 h later. The cumulus-oocyte complex was collected from the oviduct 14 h thereafter, and cumulus cells were removed by 0.1% hyaluronidase prepared in 15% fetal bovine serum in mouse embryonic fibroblast medium and then in CZB medium for 1 h to stabilize the embryo. Oocytes were then cultured for 6 h in CZB medium supplemented with 10 mmol/L SrCl_2_ and 5 μg/mL cytochalasin B to induce parthenogenetic activation. Subsequently, activated oocytes were cultured for 2 days at 37°C and 5% CO_2_ in G1 medium, and later in G2 medium. Embryonic stem cells were then harvested from developing blastocysts attached to mitomycin C-treated mouse embryonic fibroblast cells. These cells were maintained in DMEM supplemented with 15% fetal bovine serum, 1× nonessential amino acids, 1× penicillin/streptomycin/glutamine, 1× β-mercaptoethanol, and 1000 U leukemia inhibitory factor.

### Generation of biparental and parthenogenetic neural stem cells (NSCs)

Biparental and parthenogenetic neural stem cells were derived from OG2 mice, which express an Oct4-GFP transgene [19], according to methods described in our previous reports [18]. Briefly, brain tissue was collected from a 13.5 dpc (OG2^+/−^) embryo obtained by natural fertilization (and thus biparental), as well as from a 10.5-dpc parthenogenetic embryo (OG2^+/−^). Primary neurospheres were replated on gelatin-coated dishes in expansion media consisting of NSC media (Euroclone, Siziano, Italy, http://www.euroclonegroup.it) enriched with N2 supplement, 10 ng/mL each of epidermal and basic fibroblast growth factor (Invitrogen, Carlsbad, CA, http://www.invitrogen.com), 50 μg/mL bovine serum albumin (Fraction V, Gibco-BRL, Gaithersburg, MD, http://www.gibcobrl.com), 1× penicillin/streptomycin/glutamine, and 1× nonessential amino acids (Gibco BRL).

### Cell fusion

Parthenogenetic and biparental ESCs were mixed 1:1 with biparental and pNSCs, respectively, and washed in PBS. The mixture was then centrifuged at 130 ×*g* for 5 min in 50 mL conical tubes. The supernatant was discarded, and 1 mL pre-warmed 50% polyethylene glycol 1500 (Roche Diagnostics, Basel, Switzerland, http://www.roche-applied-science.com) was added dropwise to the cell pellet. DMEM was then added up to 25 mL over 5 min with constant stirring. Finally, cells were centrifuged at 130 ×*g* for 10 min, washed gently with DMEM, and seeded on a gelatin-coated dish in ES culture medium containing leukemia inhibitory factor.

### Flow cytometry

Hybrid cells were dissociated, washed with PBS, filtered through 40 μm nylon mesh, and resuspended in standard ES cell medium. Cells with highly intense GFP fluorescence were sorted directly into ES cell medium using a FACSAria cell sorter with FACSDiva software (Becton, Dickinson and Company).

### Karyotyping

Cells cultured in a 10-cm dish were treated with 3 μg/mL Nocodazole for 4 h, and digested with 0.25% trypsin/EDTA. Cells were recovered from the supernatant, treated for 15 min with a hypotonic solution (0.56% w/v KCl), and pelleted by centrifugation. Cells were then fixed and washed three times with fresh 3:1 methanol: acetic acid, and finally dropped onto clean glass slides. The slides were air-dried, stained with 4,6-diamidino-2-phenylindole (Sigma-Aldrich, St. Louis, http://www.sigmaaldrich.com), and examined under a fluorescence microscope.

### Immunocytochemistry

Cells were fixed for 20 min at room temperature with 4% paraformaldehyde, washed with PBS, and blocked for 45 min at room temperature with PBS containing 10% normal goat serum and 0.03% Triton X-100. Cells were then probed with primary antibodies against Oct4 (Oct4; monoclonal, 1:100, Abcam sc-9081), Nanog (Nanog; monoclonal, 1:200, Abcam ab80892), tubulin beta III (Tuj1; monoclonal, 1:1000, Millipore MAB1637), SMA (SMA; monoclonal, 1:200, Abcam ab7817), and Sox17 (Sox17; polyclonal, 1:200, R&D systems AF1924). Finally, cells were labeled with secondary antibodies conjugated to Alexa Fluor 488 or 568 (Molecular Probes, Eugene, OR, USA), following specifications of the manufacturer.

### Teratoma formation analysis

ES-pNSC and pES-NSC hybrid cells were harvested by dissociation solution treatment and washed twice with PBS. Prepared cells (about 10^6^) were injected into testis capsule of a severe combined immunodeficiency (SCID) mouse. After six weeks of injection, mice were sacrificed and teratomas were harvested and subjected to histophathological analysis. Dissected teratomas were fixed in 4% paraformaldehyde, processed through graded ethanol, and embedded in paraffin, followed by hematoxylin/eosin (Endoderm), PAS (Ectoderm), Alcian blue (Mesoderm) staining.

### RNA isolation and real-time RT-PCR

RNA was isolated with RNase MiniKit (Qiagen) according to the manufacturer’s protocol. cDNA was then synthesized from 1 mg total RNA using SuperScript III reverse transcriptase (Invitrogen, Grand Island, NY). For real-time PCR, standard curves were created for each target gene using known quantities of total cDNA from other cells. Target genes were amplified over 40 cycles at 95°C, 60°C, and 72°C for 30 s each, using real-time PCR primer sequences for H19 (sense, 5′-CGATTGCACTGGTTTGGA-3′ and antisense, 5′-CTCAGACGGAGATGGACGA-3′), Igf2 (sense, 5′-GGATCCCAGAACCCAAGAAGA-3′ and antisense, 5′-GGGCGGCTATTGTTGTTCTCA-3′), Peg1 (sense, 5′-CCGCGGTCCACAGTGTCGATTC-3′ and antisense, 5′-GGGGGAGGTAATACAGGGAGGCTA-3′), Peg3 (sense, 5′-TACGAATGCAAAGATTGCGGCCAG-3′ and antisense, 5′-TGGGCAGTGGCAGCTACTATTTCT-3′), and ACTB (sense, 5′-CGCCATGGATGACGATATCG-3′ and antisense, 5′ -CGAAGCCGGCTTTGCACATG-3′). ACTB was used as reference. We corrected for differences in PCR efficiency between target and reference loci using the efficiency correction in the Relative Quantification Software (Roche LC 480).

### Bisulfite genome sequencing

Genomic DNA was treated with EpiTect Bisulfite Kit (Qiagen) according to the manufacturer’s instructions, and amplified by two-step nested PCR, using bisulfite PCR primers for H19 (sense, 5′-TAAGGAGATTATGTTTATTTTTGGA-3′ and antisense, 5′-CCCCCTAATAACATTTATAACCCC-3′ for 1st round; sense, 5′-AAGGAGATTATGTTTATTTTTGGA-3′ and antisense, 5′-AAACTTAAATAACCCACAACATTACC-3′ for 2nd round), Igf2 (sense, 5′-TTTAATATGATATTTGGAGATAGTT-3′ and antisense, 5′-AAAAAACAACCTAATATAAAAAAAC-3′ for 1st round; sense, 5′-GAGTTTAAAGAGTTTAGAGAGGTTAAA-3′, and antisense, 5′-TAAACTATCCCTACTCAAAAAAAA-3′ for 2nd round), Peg1 (sense, 5′-TAGGGGTTTGTTTGTTGTTTATTT-3′ and antisense, 5′-AACCTATAAATATCTTCCCATATTC-3′ for 1st round; sense, 5′-GATATGATAGAAAATATTTTGAAATTAAAA-3′ and antisense, 5′-TAAAAATACCAACACCTAAAAAAAA-3′ for 2nd round), and Peg3 (sense, 5′-TTTTGTAGAGGATTTTGATAAGGAG-3′ and antisense, 5′-CATACTACAAACAACCAAATAACC-3′ for 1st round; sense, 5′-TGTAGAGGATTTTGATAAGGAGGTG-3′ and antisense, 5′-CAATCTAATACACCCACACTAAACC-3′ for second round). Reactions were initially denatured at 95°C for 10 min, and amplified over 45 cycles at 95°C for 60 s, 60°C for 30 s, and 72°C for 2 min, with final extension at 72°C for 10 min. Amplification products were verified by electrophoresis on 1% agarose, subcloned into pGEM-T Easy vector (Promega, Madison, WI), and sequenced with T7 primers.

### Statistical analysis

All experiments were performed in triplicate and data represented as means ± SD. Significance of differences was assessed by an unpaired t-test at p-value <0.05

## Results

### Derivation of biparental and pESCs

**Fig 1:**
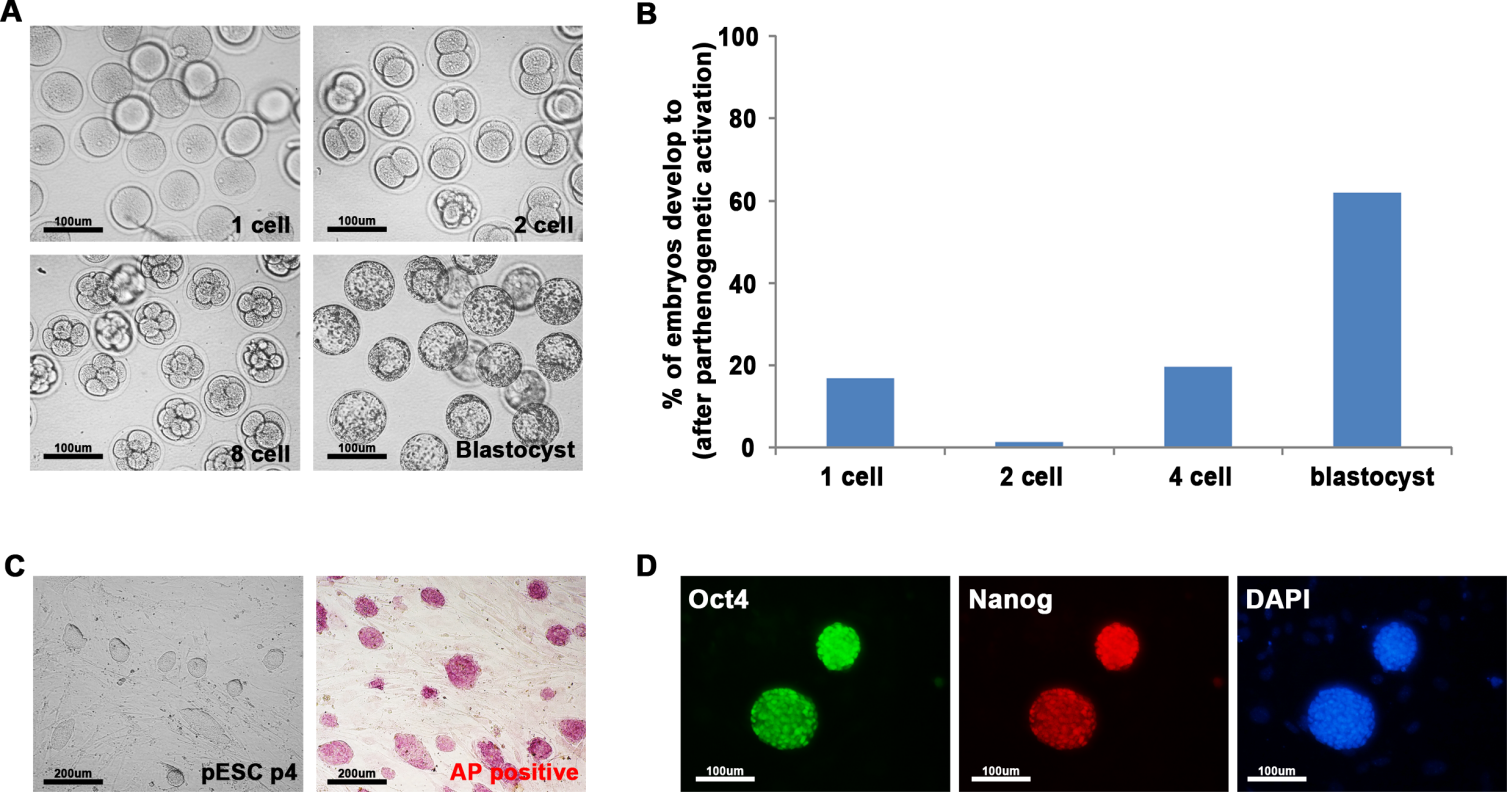
Generation of parthenogenetic ESCs (pESCs) from parthenogenetically activated embryos. **(A)** Preimplantation development of parthenogenetically activated embryos from one-cells to blastocyst stage embryos (200 ×). **(B)** Efficiency of development of parthenogenetic embryos. About 83% of oocytes were successfully activated, of which about 62% progressed to blastocyst stage. **(C)** Embryonic stem cells derived from parthenogenetic blastocysts (pESCs) were positive for the alkaline phosphatase staining (100 ×). **(D)** Immunocytochemistry of pESCs using Oct4 and Nanog antibodies (200 ×). pESCs were stained positive for key pluripotency markers, Oct4 and Nanog.

To determine whether pESCs also can reprogram somatic cells by cell-cell fusion, pESCs were newly derived and fused with NSCs. pESCs (Fig 1A) were obtained from BFD1 mice by culturing oocytes for 6 h in CZB medium containing SrCl_2_ and cytochalasin B. Approximately 62% (44/71) of parthenogenetic embryos progressed to blastocysts (Fig 1B). pESCs derived from these parthenogenetic blastocysts were morphologically similar to biparental ESCs, and expressed alkaline phosphatase (Fig 1C) and pluripotency markers such as Oct4 and Nanog (Fig 1D). These results indicate that ESCs derived from parthenogenetic blastocysts are similar to biparental ESCs.

### pESCs reprogram somatic cells, and parthenogenetic NSCs are reprogrammable by cell-cell fusion

Newly derived pESCs were fused with biparental NSCs using polyethylene glycol to test whether the former can reprogram the latter by cell-cell fusion. Conversely, parthenogenetic NSCs (pNSCs) were fused with biparental ESCs to test whether the parthenogenetic somatic cells are reprogrammable. The NSCs were established from fertilized and parthenogenetic OG2^+/-^ mouse embryos that express Oct4-GFP.

**Fig 2:**
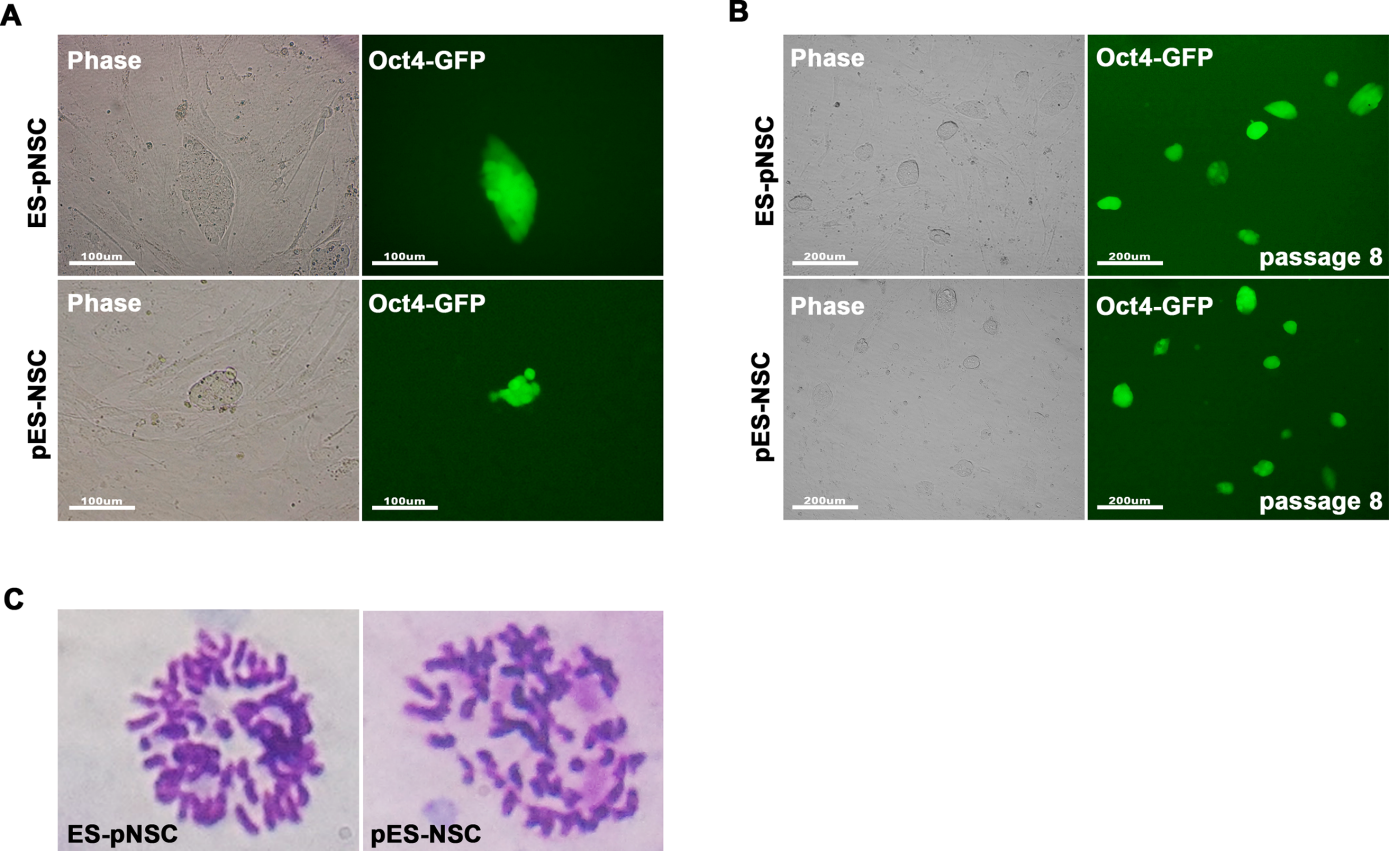
Generation of fusion hybrid cells between parthenogenetic and biparental cells. **(A)** GFP fluorescence images of fusion between biparental ESCs and parthenogenetic neural stem cells (ES-pNSC), and between pESCs and biparental neural stem cells (pES-NSC) at day 3 after fusion (200 ×). **(B)** GFP fluorescence images of ES-pNSC and pES-NSC hybrids after FACS sorting (100 ×). **(C)** Representative tetraploid karyotype of the hybrid cells.

GFP-positive (GFP^+^) cells were detected at day 3 after pESCs were fused with biparental NSCs (Fig 2A), and were established as a hybrid cell line (pES-NSC). Another hybrid cell line (ES-pNSC) was established in a similar manner using pNSCs and biparental ESCs. The hybrid cell lines were morphologically very similar to ESCs. Hybrid cells were then expanded by colony picking, re-plating in feeder-layered dishes, and sorted by FACS to obtain a pure population of reprogrammed GFP^+^ hybrid cells (Fig 2B and S1 Fig). Karyotyping showed that these cells are nearly tetraploid, confirming that they are cell fusion hybrids (Fig 2C). Parthenogenetic fusion partner cells, pESCs and pNSCs, maintained normal diploid karyotypes (S2 Fig). These results indicate that parthenogenetic pluripotent cells, pESCs, reprogram somatic cells, and parthenogenetic somatic cells, pNSCs, are reprogrammable by cell-cell fusion.

### Characterization of hybrid cells

**Fig 3:**
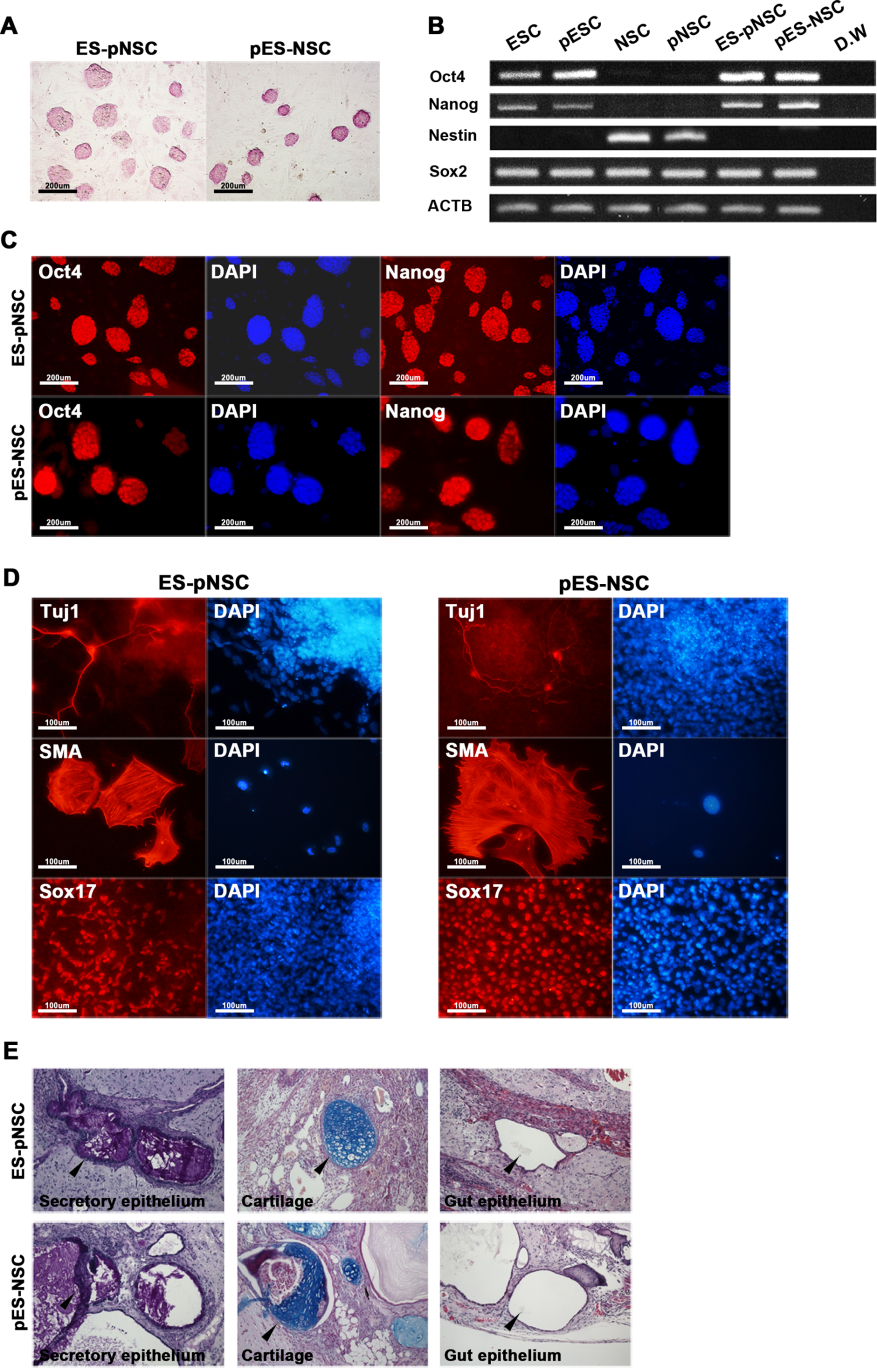
Characterization of hybrid cells. **(A)** Both ES-pNSC and pES-NSC hybrid cells are positive for alkaline phosphatase staining (100 ×). **(B)** RT-PCR analysis of *Oct4*, *Nanog*, *Sox2*, and *Nestin* expression in fusion partner and reprogrammed hybrid cells. Pluripotency markers, *Oct4* and *Nanog*, which were not expressed in NSCs and pNSCs were expressed in GFP^+^ fusion hybrid cells. On the other hand, *Nestin*, which was expressed in NSCs and pNSCs was silenced after forming GFP^+^ fusion hybrid cells. **(C)** Immunocytochemistry analysis of Oct4 and Nanog in ES-pNSC and pES-NSC hybrid cells (100 ×). **(D)** *In vitro* differentiation of ES-pNSC and pES-NSC hybrid cells into ectoderm (Tuj1), mesoderm (SMA), and endoderm (Sox17) lineages (200 ×). **(E)** In vivo differentiation potential of ES-pNSC and pES-NSC hybrid cells through teratoma assay. These hybrid cells were contributed to secretory epithelium (ectoderm), cartilage (mesoderm) and gut epithelium (endoderm), which were stained with PAS, Asian blue, and hematoxylin eosin, respectively. Each tissue was indicated by arrow head.

Hybrid cells were tested for pluripotency. Both ES-pNSC and pES-NSC hybrid cells expressed alkaline phosphatase (Fig 3A). In addition, ESCs, pESCs, and hybrid cells expressed pluripotency markers, such as *Oct4*, *Sox2*, and *Nanog*, as measured by RT-PCR (Fig 3B). In particular, *Sox2* was expressed in all cells, as it is a marker for both pluripotent cells and NSCs. *Nestin*, expressed in NSCs, was silenced after fusion-induced reprogramming. Immunocytochemistry confirmed expression of *Oct4* and *Nanog* in hybrid cells (Fig 3C). Next, hybrid cells were differentiated *in vitro* through embryoid body formation to test pluripotency. Both ES-pNSC and pES-NSC hybrid cells were differentiated into ectoderm, mesoderm, and endoderm lineages, which express Tuj1, SMA, and Sox17, respectively (Fig 3D). Teartoma assay (*in vivo* differentiation potency test) also confirmed that the fusion hybrid cells could differentiate into all three germ layers, such as ectodermal (secretory epithelium), mesodermal (cartilage) and endodermal (gut epithelium) (Fig 3E). These results suggested that NSCs or pNSCs were reprogrammed into pluripotent state by fusion with pluripotent fusion partner cells and the hybrid cells displayed pluripotent features.

### DNA methylation patterns in imprinted genes after cell-cell fusion

**Fig 4:**
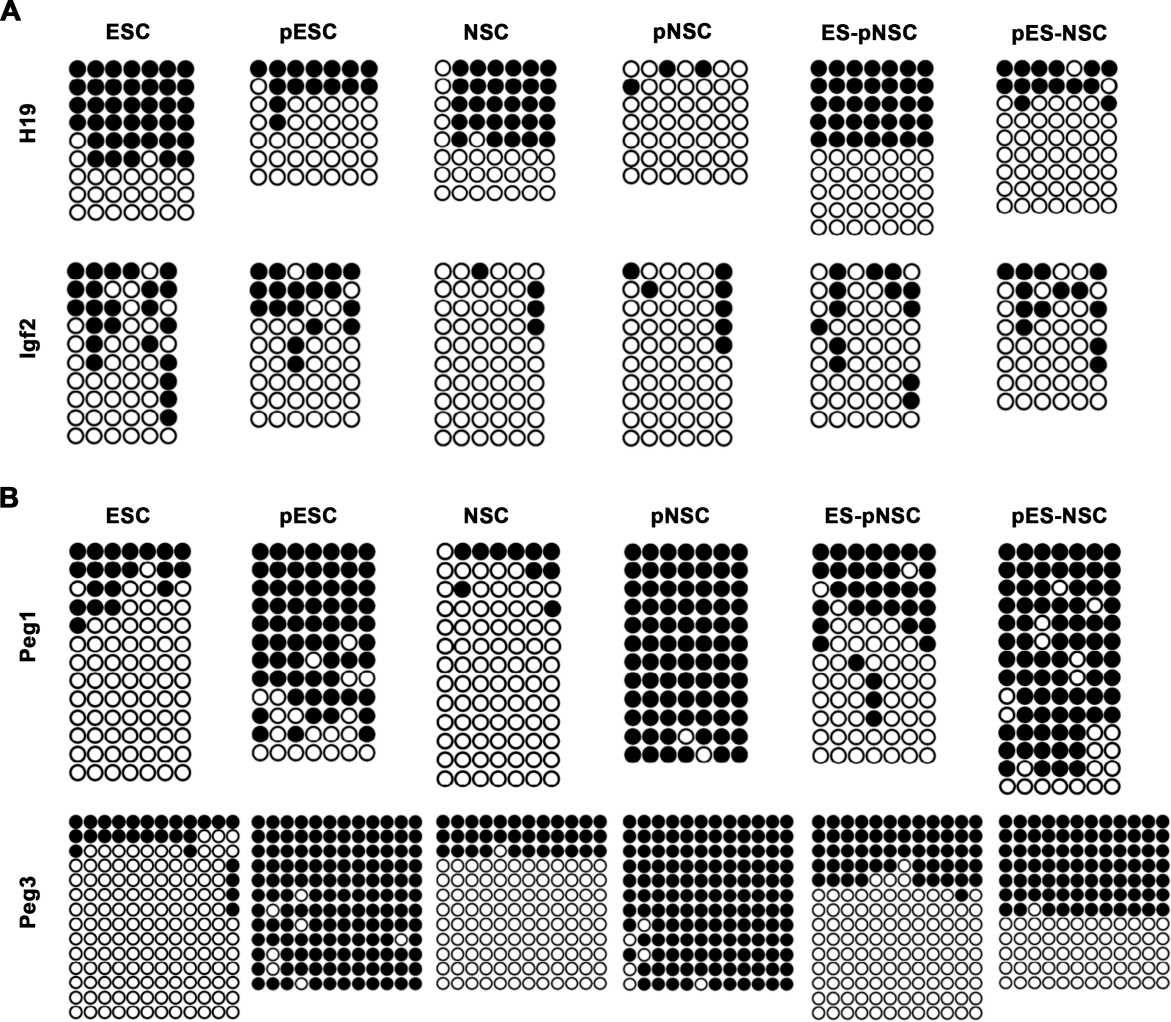
Bisulfite genome sequencing analysis of imprinted genes. DNA methylation patterns of paternally (*H19* and *Igf2*), and maternally imprinted genes (*Peg1* and *Peg3*) in ESCs, pESCs, NSCs, pNSCs, ES-pNSC, and pES-NSC hybrid cells. Black and white circles represent methylated and unmethylated CpGs, respectively.

Bisulfite genome sequencing was used to investigate possible changes in genomic imprinting patterns in hybrid cells that had been passaged more than 10 times. In particular, the paternally imprinted genes *H19* and *insulin-like growth factor 2* (*Igf2*) were analyzed, as were the maternally imprinted genes *Peg1* and *Peg3*. *H19* and *Igf2* are physically clustered in the genome, and share the same enhancers and control elements. However, *H19* is expressed only from the maternal allele (paternally imprinted and maternally expressed), while *Igf2* is expressed only from the paternal allele (paternally imprinted and paternally expressed) [20]. In accordance with our previous report [16], these genes were completely demethylated in pNSCs lacking a paternal allele, while *Peg1* and *Peg3* were completely methylated (Fig 4A and 4B). On the other hand, *H19* and *Igf2* were partially methylated in pESCs, as in biparental ESCs, but *Peg3* was completely demethylated (Fig 4A and 4B). These results indicate that parthenogenetic imprinting patterns could be altered by fusion with pESCs. Next, we tried to perform the SNP-based methylation analysis, but it cannot be possible because there was no SNP in these genes between fusion partner cells (between ESCs and pNSCs, and pESCs and NSCs) (S3 Fig).

We then investigated whether DNA methylation patterns in somatic cells are altered after fusion with pluripotent stem cells. Notably, methylation patterns of *H19* and *Igf2* in ES-pNSC hybrid cells differ from those in pNSCs, but are comparable to those in ESCs (Fig 4A). Similarly, pES-NSC hybrid cells showed similar DNA methylation patterns as pESCs. These results indicate that methylation marks on paternally imprinted genes were altered in hybrid cells to resemble those in pluripotent stem cells. In contrast, changes in methylation on the maternally imprinted genes *Peg1* and *Peg3* were not as clear as *H19* or *Igf2*. For instance, DNA methylation patterns on *Peg1* and *Peg3* in ES-pNSC hybrids were similar to those of ESCs (Fig 4B), but, in pES-NSC hybrids, methylation patterns were similar to those pESCs and NSCs, respectively. Based on these observations, it is possible that (1) imprinting patterns from pESCs and NSCs coexisted independently in pES-NSC hybrids, or (2) imprinting patterns in pESCs were modified to resemble those of biparental NSCs. In any case, these data suggest that pluripotent fusion partners do not necessarily determine the status of imprinted genes in the resulting hybrids.

**Fig 5:**
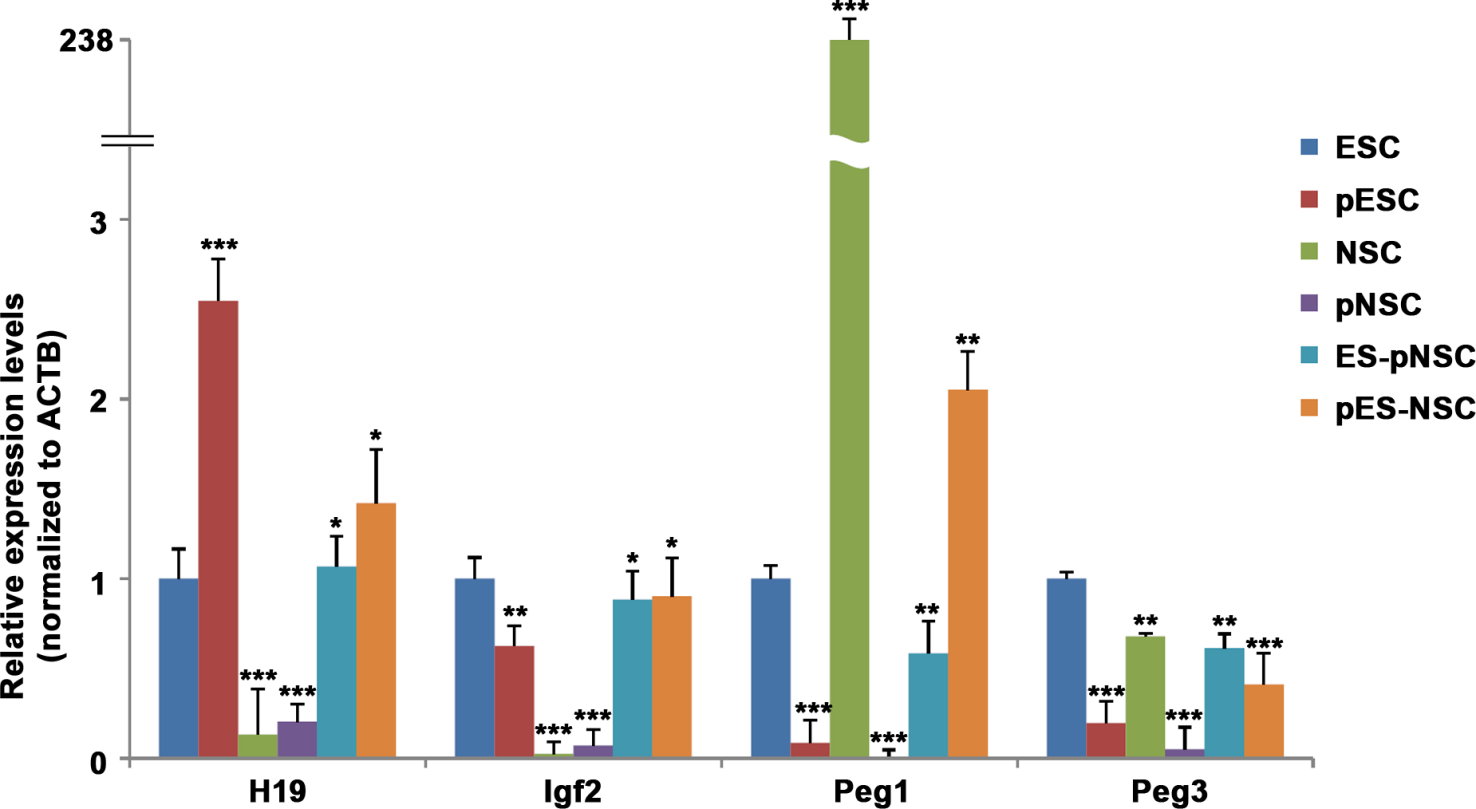
Quantitative RT-PCR analysis of imprinted gene expression. The expression profiles of paternal and maternal imprinted genes were analyzed by real-time RT-PCR. All data are normalized to *ACTB* expression and calibrated on the ESCs, whose gene expression was considered 1 for all genes. Error bars represent mean values ± SEM of three independent experiments. Student’s t-test: ***, p<0.001; **, p<0.01; *, p<0.05.

Because DNA methylation generally silences genes and demethylation arrows activation of genes, we measured the expression of the representative imprinted genes, *H19*, *Igf2*, *Peg1* and *Peg3*, by quantitative RT-PCR (Fig 5). *H19* and *Igf2* was not expressed in biparental NSCs, but was expressed at low levels in pNSCs, presumably because the gene is demethylated in both alleles (two maternal genome). In addition, *H19* was expressed in pESCs at about twice the level as that in ESCs (Fig 5), presumably as a result of reduced methylation (Fig 4A). Similarly, *H19* was twice more abundant in pES-NSC hybrids than in ES-pNSC hybrids, reflecting the pattern observed in the ESC fusion partners and the DNA methylation patterns showed in hybrids. Also, *Igf2* is more expressed in ESC and pESC than NSC and pNSC. Both fusion hybrid RNA expression level were similar to pluripotent cells. On the other hand, pESCs and pNSCs, in which *Peg1* and *Peg3* are completely methylated (Fig 4B), expressed *Peg3* at negligible levels (Fig 5). *Peg3* was also expressed negligibly in biparental NSCs, even though the gene is the normal pattern of imprinted genes (differentially methylated pattern) in these cells. Moreover, ESCs and ES-pNSC hybrids expressed *Peg3* at similar levels, while pES-NSC hybrids expressed *Peg3* much more abundantly than both pESCs and NSCs. These results indicate that regulation of *Peg3* expression does not depend solely on methylation.

## Discussion

Fusion-induced reprograming is a powerful technique that induces pluripotency in differentiated cells within 2 days, in contrast to induced pluripotency, which requires more than 7 days [21–23]. In this light, we evaluated the ability of pluripotent stem cells to reprogram and reconfigure genomic imprinting in somatic cells through cell fusion. Indeed, we previously demonstrated that the epigenetic status of somatic cells is altered to resemble that of pluripotent fusion partners [8, 18]. Accordingly, we speculated that genomic imprinting, also a type of epigenetic regulation, would be similarly modified.

To characterize changes in genomic imprinting, we fused parthenogenetic somatic and pluripotent cells with biparental pluripotent and somatic cells, respectively. We found that pESCs successfully reprogram somatic cells, and that pNSCs are reprogrammable into pluripotent cells by cell-cell fusion. In most cases, methylation patterns of imprinted genes in hybrid cells resembled those in pluripotent fusion partners. However, we could not conclude that pluripotent cells universally determine imprinting patterns in fused cells, as we cannot exclude the possibility that unaltered somatic alleles may persist along with pluripotent alleles in the tetraploid hybrid cells. Indeed, we found clear evidence that *Peg3*, a maternally imprinted and paternally expressed gene, is methylated differently in pES-NSC hybrid cells than in pESCs. However, pES-NSC hybrid cells expressed *Peg3* abundantly, in contrast to the initial two fusion partners. Remarkably, *Peg3* is also expressed abundantly in biparental ESCs, suggesting that the gene was somehow reprogrammed in pES-NSC hybrid cells to resemble its epigenetic status in biparental ESCs. Taken together, these results indicate that reprogramming does not necessarily return imprinting patterns in somatic cells to the pluripotent state. Thus, reprogramming using parthenogenetic cells may be a useful tool to investigate genomic imprinting mechanisms, as well as expression of imprinted genes.

## Supporting Information

S1 Fig FACS sorting for Oct4-GFP+ hybrid cells. High GFP+ cells (P1 box) were sorted and further cultured to maintain pure population of reprogrammed fusion hybrid cells.
(PDF)

S2 Fig Representative normal diploid karyotypes of parthenogenetic fusion partner cells. In both case, more than 90% cells examined contained normal 40 chromosomes (n = 30).
(PDF)

S3 Fig Comparison of DNA sequences between fusion partner cells in H19, Ifg2, Peg1, and Peg3 genes. The sequences between ESCs and pNSCs and between pESCs and NSCs were 100% identical in H19, Ifg2, Peg1, and Peg3 genes. Thus, there was no SNP in these genes between fusion partner cells (between ESCs and pNSCs, and pESCs and NSCs). These analyses were performed using BLAST® program of National Library of Medicine.
(PDF)
