# Supplementary material for: Changes in Parthenogenetic Imprinting Patterns during Reprogramming by Cell Fusion
Source: PLoS One. 2016 May 27;11(5):e0156491. doi: 10.1371/journal.pone.0156491 (PMC4883797; doi:10.1371/journal.pone.0156491)
Supplement: S1 Fig — High GFP+ cells (P1 box) were sorted and further cultured to maintain pure population of reprogrammed fusion hybrid cells. (PDF) [file pone.0156491.s001.pdf]

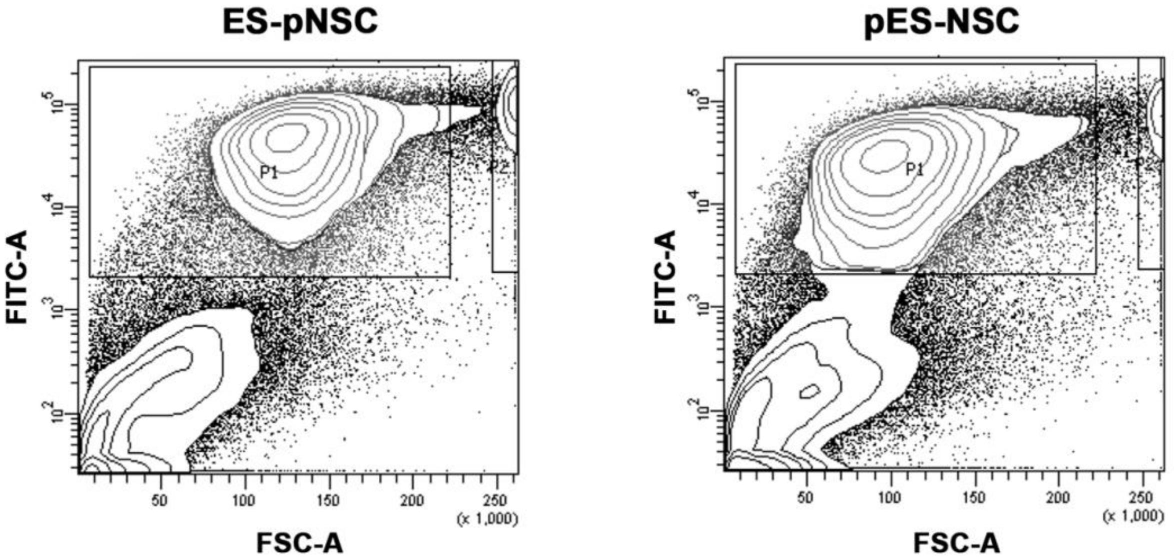

Fig S1. FACS sorting for Oct4-GFP⁺ hybrid cells. High GFP⁺ cells (P1 box) were sorted and further cultured to maintain pure population of reprogrammed fusion hybrid cells.
