# Supplementary material for: Changes in Parthenogenetic Imprinting Patterns during Reprogramming by Cell Fusion
Source: PLoS One. 2016 May 27;11(5):e0156491. doi: 10.1371/journal.pone.0156491 (PMC4883797; doi:10.1371/journal.pone.0156491)
Supplement: S2 Fig — In both case, more than 90% cells examined contained normal 40 chromosomes (n = 30). (PDF) [file pone.0156491.s002.pdf]

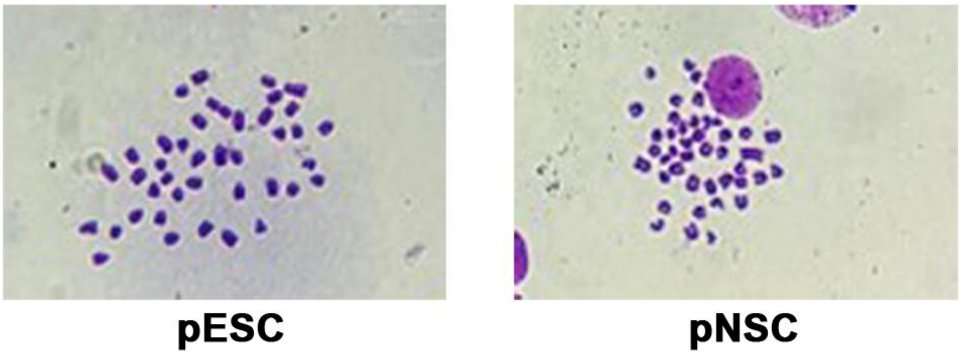

Fig. S2. Representative normal diploid karyotypes of parthenogenetic fusion partner cells. In both case, more than 90% cells examined contained normal 40 chromosomes (n=30).
