# Supplementary material for: Changes in Parthenogenetic Imprinting Patterns during Reprogramming by Cell Fusion
Source: PLoS One. 2016 May 27;11(5):e0156491. doi: 10.1371/journal.pone.0156491 (PMC4883797; doi:10.1371/journal.pone.0156491)
Supplement: S3 Fig — The sequences between ESCs and pNSCs and between pESCs and NSCs were 100% identical in H19, Ifg2, Peg1, and Peg3 genes. Thus, there was no SNP in these genes between fusion partner cells (between ESCs and pNSCs, and pESCs and NSCs). These analyses were performed using BLAST® program of National Library of Medicine. (PDF) [file pone.0156491.s003.pdf]

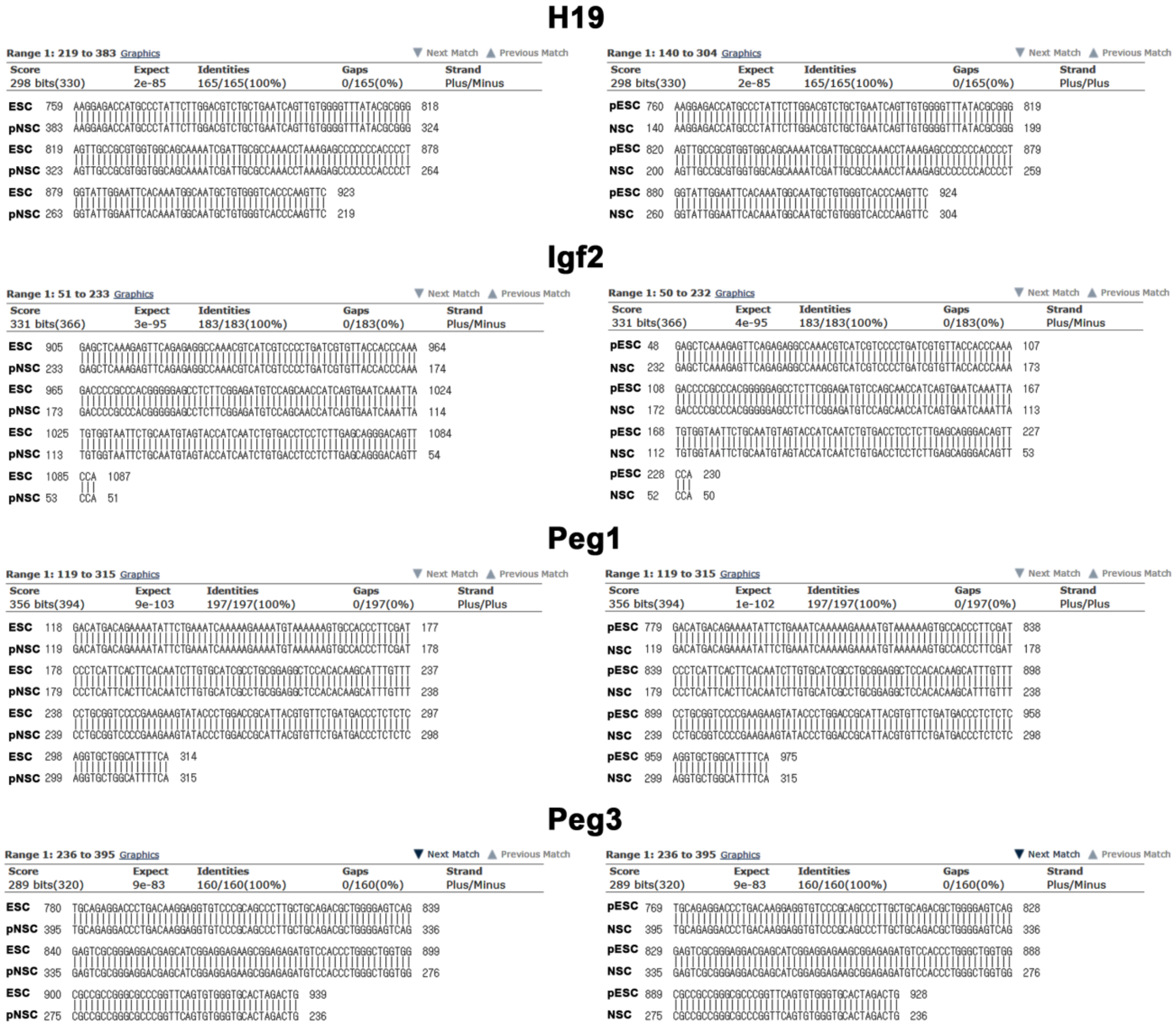

Fig. S3. Comparison of DNA sequences between fusion partner cells in H19, Ifg2, Peg1, and Peg3 genes.

The sequences between ESCs and pNSCs and between pESCs and NSCs were 100% identical in H19, Ifg2, Peg1, and Peg3 genes. Thus, there was no SNP in these genes between fusion partner cells (between ESCs and pNSCs, and pESCs and NSCs). These analyses were performed using BLAST® program of National Library of Medicine.
